# Retraction Note to: MiR-34a inhibitor protects mesenchymal stem cells from hyperglycaemic injury through the activation of the SIRT1/FoxO3a autophagy pathway

**DOI:** 10.1186/s13287-021-02606-0

**Published:** 2021-10-07

**Authors:** Fengyun Zhang, Fei Gao, Kun Wang, Xiaohong Liu, Zhuoqi Zhang

**Affiliations:** 1 Department of Cardiology, the Affiliated Hospital of Xuzhou Medical University, 99 West Huaihai Road, Xuzhou, 221000 People’s Republic of China; 2 Department of Cardiology, Institute of Cardiovascular Research, Affiliated Hospital of Xuzhou Medical University, Xuzhou, People’s Republic of China; 3 Department of Cardiology, First People’s Hospital of Suqian, Suqian, People’s Republic of China

## Retraction Note to: Stem Cell Research & Therapy (2021) 12:115 10.1186/s13287-021-02183-2

The authors have retracted this article. After publication the authors became aware of issues with two of the figures, specifically:

- In Fig. 5c the echocardiography images for 1 week and 3 weeks for the MI + HG group had been duplicated owing to an error in the assembly of the figure.
- In Fig. 3a the bands for Beclin 1 had been constructed by using the bands from P21 in Fig. 2e during assembly of the figure.

All authors agree with this retraction.

